# Construction of PEGMC Copolymerized Modified Hydrogel and Its Mechanism for Salt Retardation and Nutrient Immobilization in Dryland Soil

**DOI:** 10.3390/gels12070595

**Published:** 2026-07-03

**Authors:** Jianwei Cheng, Rui Xiang, Jingcai Liu, Baocun Yang, Xiaobing Ma

**Affiliations:** 1 College of Water Resources and Architecture Engineering, Tarim University, Aral 843300, Chinataruybc@163.com (B.Y.); 2 Chinese Research Academy of Environmental Sciences, Beijing 100012, China; 3 College of Chemistry and Chemical Engineering, Northwest Normal University, Lanzhou 730070, China

**Keywords:** polyethylene glycol maleate citrate, copolymer modification, hydrogel, dry land soil, salt retardation, nutrient immobilization

## Abstract

Aiming at severe soil secondary salinization, poor water retention and insufficient salt tolerance of conventional acrylic-based modifiers in arid and semi-arid regions of China, a poly(ethylene glycol) maleate citrate (PEGMC) crosslinking monomer was synthesized through esterification, and a dual covalent–hydrogen crosslinked P(PEGMC/AA) hydrogel was fabricated via free radical copolymerization with acrylic acid (AA). The hydrogel was characterized by NMR, FTIR, SEM, TGA and elemental mapping, while its binding mechanism with saline–alkali ions was elucidated through DFT calculations and molecular dynamics simulations. Its amelioration performance was evaluated through swelling, soil water retention, desalination and pot germination experiments. The hydrogel exhibited outstanding water absorbency, salt resistance and dry–wet cycling stability, with swelling ratios of 712 g/g in deionized water and 285 g/g in 0.9% NaCl solution, and remained 200 g/g after four dry–wet cycles. It enhanced soil water retention remarkably (over 93% after 72 h). At 0.30% dosage, soil salt content declined from 7.1 g/kg to 1.3 g/kg with desalination efficiency exceeding 80%, owing to porous physical adsorption and chemical chelation toward Na^+^, Ca^2+^ and Mg^2+^, with a binding energy of −136.936 kJ/mol. Pot tests revealed that crop germination rate rose from 19% (blank) to 75% under severe saline–alkali stress. Meanwhile, the hydrogel inhibited nutrient leaching and favored soil-water conservation. This work first incorporated PEGMC monomer into agricultural hydrogels to construct a stable dual crosslinked network, clarifying its synergistic mechanisms for salt fixation and water retention macroscopically and microscopically. It provides a promising functional material and theoretical basis for green, efficient in situ amelioration of dryland saline–alkali soil.

## 1. Introduction

Arid and semi-arid regions are an important component of inland Asia, and also one of the critical zones with the most fragile ecosystems and the most prominent human–land conflicts under global climate change [1]. This region is characterized by scarce precipitation, strong evaporation, and large diurnal temperature differences. The unique coupled water–salt transport drives the continuous upward movement of groundwater and deep soil water through capillary pores, leading to the persistent accumulation of soluble basic ions in the topsoil and forming a typical surface-accumulated salinization profile. This severely disrupts soil aggregate structure, reduces pore connectivity, and inhibits microbial activity and nutrient transformation, resulting in a continuous decline in land productivity, difficulties in vegetation restoration, and highly unstable agricultural yields [2]. At the same time, dryland soils generally suffer from insufficient organic matter, low colloid content and weak retention capacity. Exogenous nutrients are easily leached deeply with instantaneous precipitation or irrigation water, which not only greatly reduces fertilizer utilization efficiency, restricts seed germination and seedling establishment, but also causes risks of non-point source pollution and excessive nitrate nitrogen in groundwater [3,4]. Therefore, developing novel soil-regulating materials with integrated functions of water retention, salt–alkali inhibition, nutrient immobilization, and synergistic soil improvement has become an urgent scientific issue to be addressed in the efficient utilization of water and soil resources, remediation of degraded soils, and ecological restoration in arid regions.

Polymeric hydrogels show irreplaceable potential for agricultural and forestry water retention, soil and water conservation, and saline–alkali soil amelioration, owing to their 3D hydrophilic networks, ultrahigh water absorbency, and excellent structural designability. They are considered one of the key functional materials for addressing water–salt imbalance and nutrient loss in dryland soils [5]. Among them, acrylic-acid-based superabsorbent polymers have become the mainstream category of agricultural water-retaining agents owing to their accessible raw materials, simple synthesis, and outstanding water absorption performance [6]. Alsharaeh et al. [7] systematically confirmed that polyacrylic acid/polyacrylamide hydrogels can increase the water-holding capacity of sandy soil by several times and maintain a stable water-absorbing morphology even under extreme high temperature and drought conditions, demonstrating excellent potential for water and soil regulation. However, traditional single-component acrylic acid hydrogels have inherent drawbacks such as simple cross-linked structure, brittle network, poor thermal stability, and weak tolerance to drying–wetting cycles. In the complex dryland environment characterized by drying–wetting alternation, drastic temperature variation, and salt stress, these hydrogels are prone to network collapse, abrupt decay of water absorption performance, structural pulverization and failure [7]. More importantly, conventional acrylic acid gels only possess physical water storage capacity, lacking active inhibition on salt ion migration. They cannot suppress the typical capillary salt accumulation process in drylands, nor can they achieve long-term retention of nutrients such as nitrogen, phosphorus and potassium, which greatly limits their long-term application in soils under combined saline–alkali stress [8].

To address the bottlenecks of single functionality and insufficient stability in traditional hydrogels, researchers have conducted extensive studies on copolymerization modification, composite reinforcement, and structural optimization. Wu et al. [9] prepared a CMC-based semi-interpenetrating network hydrogel that achieved sustained nutrient release for over 30 days in saline–alkali soil, enhanced soil enzyme activity, and decreased soil pH. However, its swelling stability in high-salt environments still requires further improvement. In terms of inorganic nanocomposites, the introduction of biochar, kaolin and other fillers into double-network hydrogels has significantly enhanced the salt tolerance and cycling stability of the materials, but problems such as easy agglomeration of inorganic phases and weak interfacial bonding have not been fundamentally solved [10,11]. In the aspect of functional monomer copolymerization, monomers such as 2-acrylamido-2-methylpropanesulfonic acid (AMPS) and maleic anhydride have been widely used to modify acrylic acid systems, which can effectively introduce salt-resistant groups and optimize network structures. However, most existing modifications only focus on the improvement of a single property, making it difficult to simultaneously achieve the synergistic enhancement of the three core functions of water retention, salt resistance, and nutrient fixation [12].

PEGMC is a multifunctional hydrophilic unsaturated polyester monomer synthesized via esterification of CA, PEG-2000. Its molecular structure integrates flexible polyether long chains, highly reactive carboxyl groups, ester groups and polymerizable carbon–carbon double bonds, endowing it with multiple advantages including multifunctional cross-linking, strong hydrophilicity, ion complexation and biocompatibility [13]. PEG-based hydrogels have been extensively studied in biomedical fields such as drug delivery, proving their excellent biocompatibility and structural tunability [14]. The temperature-sensitive PEG-based hydrogel developed by Tran’s group (2025) can form a gel in situ at body temperature and exhibits antioxidant properties, demonstrating the unique synergistic effect of multiple functional groups [14]. However, current research on PEG-based hydrogels is highly concentrated in the biomedical field, and specific studies targeting water–salt regulation and nutrient retention in dryland saline–alkali soils remain blank. As a novel esterified functional monomer, the copolymerization mechanism of PEGMC with acrylic acid systems, the evolution law of its microstructure, and the structure–activity relationship between “structure–performance–soil improvement effect” have not been systematically reported. Meanwhile, most existing amendment materials focus on single-function optimization, lacking research on the synergistic regulation mechanism of the coupled water–salt–nutrient transport process in drylands, which makes it difficult to realize long-term remediation of degraded soils and improvement of soil fertility.

Based on the above research background and existing technical bottlenecks, this study intends to prepare PEGMC-modified acrylic composite functional hydrogels via free radical copolymerization. Through molecular structure design and cross-linked network optimization, the synergistic improvement of thermal stability, drying–wetting cycle resistance, ion barrier ability and nutrient retention performance of the materials will be achieved simultaneously. Characterization techniques including Fourier transform infrared spectroscopy (FT-IR), nuclear magnetic resonance spectroscopy (NMR), thermogravimetric analysis (TG), and scanning electron microscopy (SEM) will be adopted to systematically reveal the regulatory mechanism of PEGMC copolymerization modification on the chemical structure, bonding mode, thermal stability and micromorphology of the hydrogels. Focusing on the actual habitat of drylands, the water retention and moisture stabilization properties as well as repeated drying–wetting swelling stability of the materials under different temperatures, soil textures and salt gradients will be investigated. By simulating the capillary salt accumulation process of groundwater, the barrier mechanism of the hydrogel on the migration of main soil salt ions (Na^+^, K^+^, Ca^2+^, Mg^2+^) and the regulation law of surface soil electrical conductivity will be clarified. Combined with seed germination and pot experiments, the improvement effects of the modified hydrogel on dryland soil habitat optimization, nutrient retention and crop stress resistance will be evaluated. This study aims to reveal the synergistic mechanism of salt inhibition and nutrient fixation of PEGMC copolymerized modified hydrogels, so as to provide novel polymer functional materials and theoretical support for the improvement of saline–alkali degraded soils, efficient utilization of water and fertilizer resources, and ecological restoration in arid and semi-arid regions [13].

## 2. Results and Discussion

### 2.1. Molecular Structure Characterization Through ^1^H NMR

**Figure 1:**
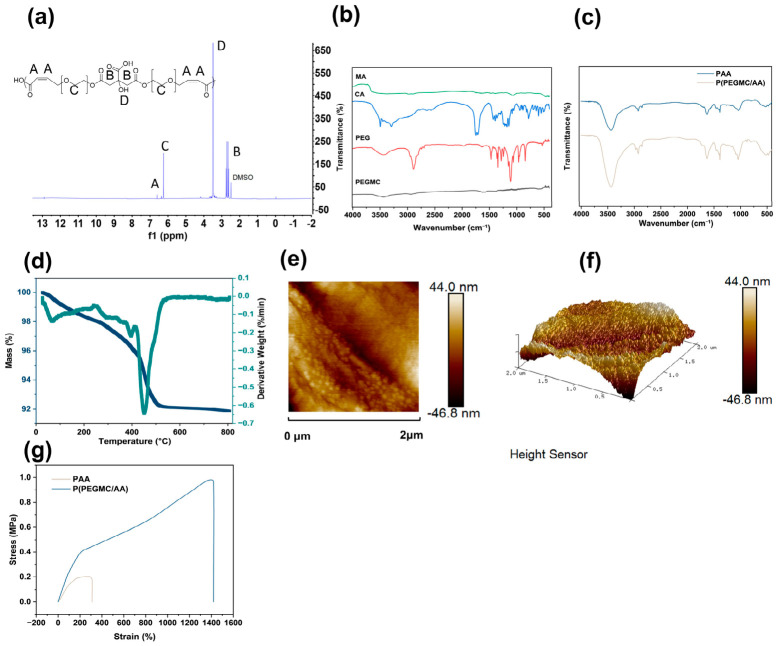
Systematic characterization of synthesized crosslinker and as-prepared hydrogels. (**a**) ¹H NMR spectrum of PEGMC crosslinker recorded in DMSO-d₆ (A: δ = 6.5–6.7 ppm; B: δ = 2.5–3.0 ppm; C: δ = 3.5–3.7 ppm; D: δ = 3.3–3.6 ppm). The signal at δ = 2.5 ppm corresponds to DMSO-d₆ solvent. (**b**) FT-IR spectra of raw materials MA, CA, PEG and synthesized PEGMC; (**c**) FT-IR comparison of pure PAA and P(PEGMC/AA) composite hydrogel; (**d**,**e**) 2D and 3D AFM surface topography images of P(PEGMC/AA) hydrogel; (**f**) TG and DTG curves of PAA and P(PEGMC/AA) hydrogels; (**g**) stress–strain tensile curves of different hydrogel samples.

The ^1^H NMR spectrum of the PEGMC crosslinker is shown in Figure 1a. The characteristic proton peaks were precisely assigned according to the molecular structure as follows:

The characteristic peak at chemical shift δ ≈ 6.5–6.7 ppm (peak A) was attributed to the unsaturated double-bond protons on the maleic acid moiety. The intense peak at δ ≈ 6.2–6.4 ppm (peak C) corresponded to the repeated -O-CH_2_CH_2_- methylene protons in the PEG long-chain backbone. The multiple peaks in the range of δ ≈ 2.5–3.0 ppm (peak B) originated from the aliphatic methylene protons on the citric acid skeleton. The peak D is assigned to the hydroxyl-substituted methine proton of citric acid units. The strong peak near δ ≈ 2.5 ppm was the characteristic peak of DMSO solvent.

All target characteristic proton signals in the ^1^H NMR were completely matched and clearly distinguishable, while the impurity signals of unreacted free monomers were significantly weakened. This directly verified that the esterification and grafting reaction among citric acid, PEG2000 and maleic anhydride was successfully carried out, and the functional PEGMC crosslinker with long flexible chains, multiple carboxyl active sites and terminal polymerizable double bonds was obtained, which provided sufficient reactive sites for the subsequent free radical crosslinking copolymerization with acrylic acid monomers [13].

### 2.2. Functional Group Verification Through Fourier Transform Infrared Spectroscopy (FT-IR)

The FT-IR spectra of raw materials MA, CA, PEG and the synthesized PEGMC crosslinker are shown in Figure 1b, and the infrared comparison of PAA and P(PEGMC/AA) composite hydrogel is presented in Figure 1c.

Compared with the spectra of MA, CA and PEG monomers, the PEGMC spectrum shows a significantly enhanced characteristic absorption peak of ester C=O stretching vibration at 1720 cm^−1^, while the intensity of the hydroxyl O-H characteristic peak near 3400 cm^−1^ is greatly weakened. This directly confirms the esterification and dehydration reaction between carboxyl and hydroxyl groups, and the successful formation of covalent ester bonds. The ^1^H NMR and FT-IR results corroborate each other, further verifying the successful synthesis of the PEGMC crosslinker [13].

Compared with the PAA hydrogel, the broad bands of –OH and –COOH groups of the P(PEGMC/AA) composite hydrogel at 3200–3600 cm^−1^ show obvious redshift and broadening, suggesting the formation of numerous intermolecular and intramolecular hydrogen bonds in the composite system. Meanwhile, the characteristic absorption peak of the acrylic C=C double-bond near 1640 cm^−1^ almost completely disappears, confirming efficient free radical copolymerization between the terminal double bonds of PEGMC and acrylic acid monomers, and the successful construction of the 3D covalently crosslinked network.

These results confirm the successful preparation of the composite hydrogel from the perspective of chemical bonds and functional groups. Abundant polar groups (carboxyl, hydroxyl, etc.) remaining on the material surface also provide a structural basis for its excellent water retention, salt ion complexation, and saline–alkali soil amelioration performance.

### 2.3. AFM Microscopic Surface Morphology Analysis

Atomic force microscopy (AFM) was used to characterize the surface microscopic morphology of the P(PEGMC/AA) composite hydrogel. The 2D and 3D AFM images are presented in Figure 1d and Figure 1e, respectively, with a scanning area of 2 μm × 2 μm. The overall height fluctuation of the sample surface ranged from −46.8 to 44.0 nm.

AFM results reveal that the as-prepared P(PEGMC/AA) hydrogel exhibits a non-smooth but uniform, fine surface morphology with a dense and regular structure and no obvious large defects or cracks. This rough microscopic surface significantly increases the specific surface area of the hydrogel. On the one hand, it provides more physical sites for water adsorption, storage, and slow release, greatly enhancing the long-term water absorbency and retention of the material. On the other hand, it fully exposes active functional groups within the gel, strengthens the adsorption and complexation of free salt cations in soil, effectively suppresses topsoil salt accumulation, and provides morphological support for the long-term amelioration of saline–alkali soil [15].

### 2.4. Thermal Stability Analysis (TG/DTG)

Thermogravimetric (TG) and derivative thermogravimetric (DTG) curves of PAA and P(PEGMC/AA) hydrogels are shown in Figure 1f to evaluate the thermal decomposition behavior and thermal structural stability of different hydrogel systems [16].

The overall thermal weight loss process of hydrogels can be divided into three typical stages:

25–200 °C: slight mass loss, mainly due to the evaporation of physically adsorbed free water and bound water inside the material [17];

200–450 °C: rapid mass loss, corresponding to the thermal oxidative decomposition of polar functional groups such as carboxyl and ester bonds on the hydrogel side chains [18];

450–600 °C: main thermal degradation stage, in which the three-dimensional crosslinked polymer backbone breaks and undergoes complete pyrolysis and carbonization [19].

Compared with pure PAA hydrogel, the P(PEGMC/AA) composite hydrogel exhibits significantly higher initial thermal decomposition temperature, temperature at maximum weight loss rate, and higher char residue at high temperatures. These results confirm that the dual network structure of “covalent crosslinking + hydrogen bonding” constructed by PEGMC long-chain crosslinking modification significantly enhances the interaction between polymer chains and the stability of the overall network, greatly improving the thermal stability of the material. This enables the material to maintain stable structure and function for a long time in complex and variable field soil environments [20].

### 2.5. Mechanical Tensile Properties Analysis

The stress–strain curves of PAA and P(PEGMC/AA) composite hydrogels are shown in Figure 1g.

The pure PAA hydrogel possesses a single crosslinked network with limited internal energy dissipation pathways, resulting in brittle mechanical performance. It exhibits a low elongation at break of approximately 250% and a tensile strength of merely 0.22 MPa, rendering it susceptible to fracture under external force.

Incorporation of the flexible long-chain PEGMC crosslinker dramatically improves the mechanical properties of the P(PEGMC/AA) composite hydrogel. Its elongation at break exceeds 1400%, and the maximum tensile strength approaches 1.0 MPa, indicating a several-fold enhancement in material toughness and load-bearing capacity.

Mechanistically, the introduced flexible PEGMC long chains construct an efficient reversible energy dissipation system within the hydrogel network. Under external tensile deformation, reversible hydrogen bonds preferentially rupture and dissipate external energy, while the covalently crosslinked backbone maintains the integral network structure. This synergistic effect greatly enhances the deformation and fracture resistance of the composite hydrogel.

These superior mechanical properties enable the hydrogel to resist structural failure under practical field conditions, including repeated soil drying–wetting cycles, external extrusion, and plant root penetration. Accordingly, the material can sustain stable water retention and salt reduction functions for long-term soil amelioration [14].

### 2.6. Scanning Electron Microscopy (SEM) and Elemental Distribution Characterization

**Figure 2:**
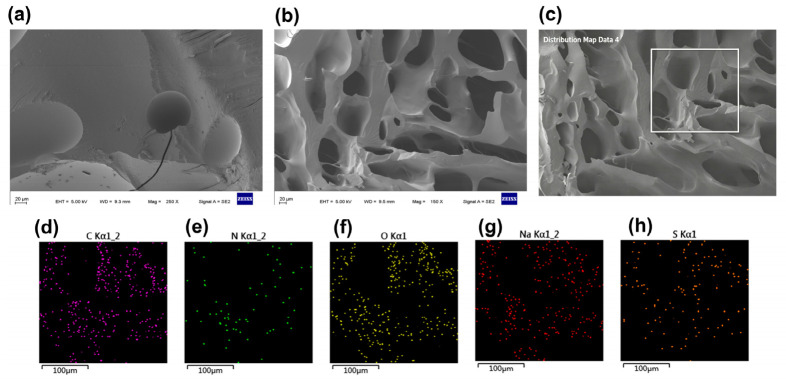
Microstructure morphology and elemental distribution of different hydrogel samples. (**a**) SEM image of pure PAA hydrogel; (**b**,**c**) cross-sectional SEM morphology of P(PEGMC/AA) composite hydrogels with different ratios; the bottom shows the typical elemental mapping results of P(PEGMC/AA) composite hydrogel; (**d**–**h**) Corresponding elemental mapping of C, N, O, Na and S, respectively, obtained from the framed area in panel (**c**).

SEM cross-sectional morphologies of pure PAA and modified P(PEGMC/AA) hydrogels are presented in Figure 2a–c. The pure PAA hydrogel (Figure 2a) shows a dense structure with few irregular pores and limited surface protrusions, exhibiting a poorly developed 3D interconnected network. After PEGMC copolymerization, the P(PEGMC/AA) hydrogels (Figure 2b,c) form abundant, uniformly distributed, interconnected hierarchical pores. The modified hydrogels possess thin, continuous pore walls and greatly improved pore connectivity. Mechanistically, the flexible long PEGMC chains and rich polar functional groups regulate the polymerization crosslinking density and phase separation behavior, thereby inducing the in situ formation of a stable porous framework [21]. This well-developed and interconnected pore structure provides sufficient storage space for the rapid adsorption, retention and long-term slow release of water molecules, greatly improving the swelling ability of the material and soil water retention capacity.

At the same time, it greatly increases the specific surface area of the material, fully exposes the abundant carboxyl and hydroxyl active sites on the gel surface, significantly enhances the complexation and adsorption performance for salt ions such as Na^+^, Ca^2+^ and Mg^2+^ in soil, and strengthens the saline–alkali barrier effect [21].

The corresponding elemental mapping images clearly confirm the uniform distribution of C, O, Na, Ca, Mg and other characteristic elements in the composite hydrogel framework, further demonstrating the homogeneous dispersion of functional groups and active sites throughout the material after PEGMC modification.

These results also verify the excellent immobilization and complexation ability of the hydrogel toward saline–alkali cations, providing direct microscopic evidence for its long-term application in soil amelioration [22].

### 2.7. Swelling Response Behavior and Salt-Resistant Water Retention Performance of Hydrogels

**Figure 3:**
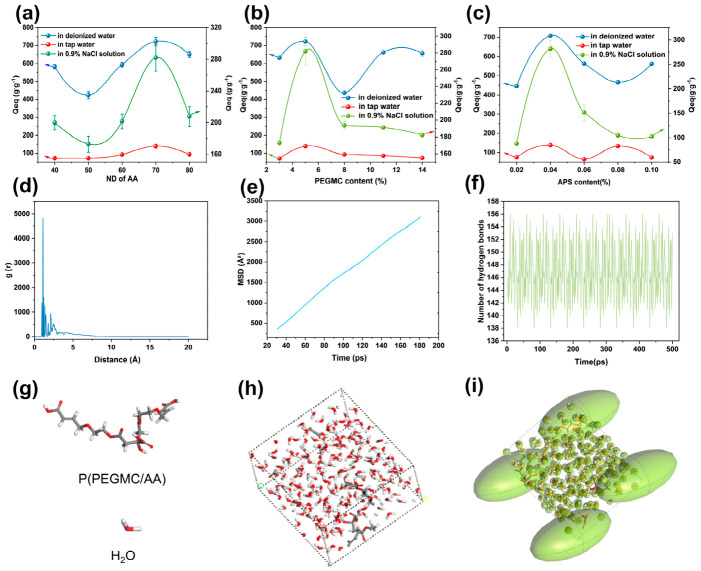
Swelling properties and molecular dynamics simulation of hydrogel. (**a**) Effect of neutralization degree; (**b**) effect of PEGMC content; (**c**) effect of APS dosage; (**d**) radial distribution function; (**e**) MSD of water molecules; (**f**) variation of hydrogen bond number; (**g**) molecular model; (**h**) initial simulation configuration, A, B and C refer to three marked observation positions in the periodic simulation box; (**i**) molecular surface distribution for hydrogen bond analysis, A, B and C refer to three marked observation positions on the molecular aggregate surface.

Figure 3a–c show the effects of neutralization degree of acrylic acid, dosage of PEGMC crosslinker, and dosage of APS initiator on the equilibrium swelling ratio of P(PEGMC/AA) composite hydrogels in deionized water, tap water, and 0.9% NaCl solution, respectively.

Overall, the swelling ratio of the hydrogels in the three media first increases and then decreases with increasing component contents. The swelling performance reaches its maximum at the optimal neutralization degree, PEGMC dosage, and APS dosage. The optimized hydrogel exhibits an equilibrium swelling ratio of 712 g/g in deionized water, above 120 g/g in tap water, and still 285 g/g in 0.9% saline solution, demonstrating excellent salt tolerance.

Mechanistically, neutralization of acrylic acid using NaOH generates abundant carboxylate ions (–COO^−^) along the polymer chains. With increasing neutralization degree, the negative charge density and electrostatic repulsion within the network increase, causing the polymer chains to stretch fully and the 3D network to expand, thus accommodating more water and increasing the macroscopic swelling ratio. Meanwhile, the abundant polar groups form strong hydrogen bonds with water molecules, further enhancing hydrophilicity. However, an excessively high neutralization degree increases the system’s ionic strength and strengthens the charge shielding effect, which restricts chain stretching and shrinks network pores, eventually reducing the swelling capacity [23].

Appropriate incorporation of PEGMC long-chain crosslinker and APS initiator effectively regulates the crosslinking density of hydrogel networks. On the one hand, it introduces additional hydrophilic sites (hydroxyl and ether groups) to synergistically improve water absorption capacity. On the other hand, it constructs a structurally stable 3D network that prevents excessive dissolution and structural collapse of the hydrogel in aqueous environments. Nevertheless, excessive dosages of crosslinker and initiator lead to overly high crosslinking density, which hinders polymer chain movement, reduces network porosity, and ultimately weakens the hydrogel swelling performance [24].

Compared with pure PAA hydrogel, the swelling performance of this system decreases less in salt solution. The synergistic effect formed by multiple polar functional groups and charged groups inside the material can effectively resist the osmotic pressure change and charge shielding effect caused by external salt ions, endowing the material with excellent salt-resistant swelling stability, which is fully suitable for the complex water environment of saline–alkali soil and lays a performance foundation for long-term water retention.

### 2.8. Elucidation of Water Retention Mechanism Through Molecular Dynamics

Molecular dynamics simulations were performed to reveal the water absorption, salt tolerance, and water retention mechanisms of the composite hydrogel at the molecular level, as shown in Figure 3d–i.

The radial distribution function (Figure 3d) shows strong short-range peaks between P(PEGMC/AA) chains and water molecules, indicating the formation of strong hydrogen bonds between polar/carboxylate groups of the polymer and water, with significant intermolecular binding.

The mean square displacement (MSD) curves of water molecules (Figure 3e) reveal a much lower MSD growth rate than that of free water, demonstrating that the gel network significantly restricts water migration and diffusion, thereby effectively slowing water flow and evaporation loss.

The hydrogen bond number evolution (Figure 3f) indicates that the number of hydrogen bonds remains stable and high throughout the simulation, forming a dynamically stable network that tightly immobilizes water molecules within the gel framework.

From the molecular models and simulation configurations (Figure 3g–i), the neutralized polymer chains combined with flexible PEGMC segments clearly construct a 3D confinement network that anchors water via electrostatic interactions and hydrogen bonding.

Macroscopic swelling tests and microscopic simulations mutually verify the intrinsic mechanism responsible for the high water absorbency, strong salt resistance, and long water retention of the composite hydrogel, endowing it with clear advantages for soil moisture conservation and saline–alkali land amelioration.

### 2.9. Soil Water Retention, Nutrient Slow Release and Environmental Adaptability of Composite Hydrogels

**Figure 4:**
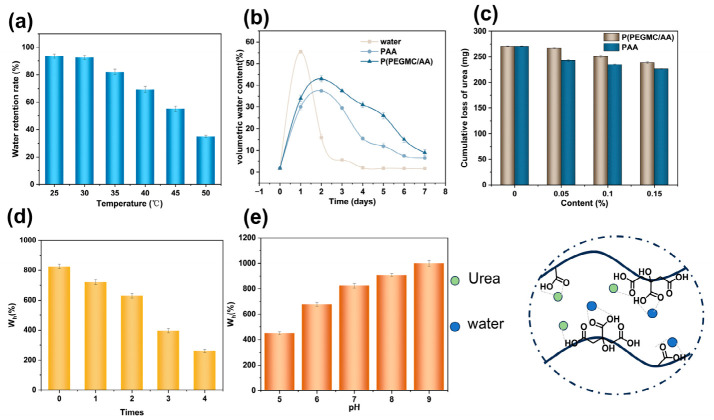
Water retention and soil amendment performance of P(PEGMC/AA) composite hydrogel. (**a**) Water retention rate of hydrogel under different temperatures; (**b**) temporal variation of volumetric water content of blank, pure PAA and P(PEGMC/AA) amended soil; (**c**) cumulative water loss of soil with different hydrogel dosages; (**d**) repeated water retention cycling stability of P(PEGMC/AA) hydrogel; (**e**) water and urea nutrient retention capacity of hydrogel at different pH values. The inset shows the interaction mechanism between hydrogel network, water and urea molecules.

In this section, the long-term water retention, soil moisture regulation, fertilizer slow release and reusability of P(PEGMC/AA) composite hydrogels under various environmental conditions were systematically investigated, and the results are shown in Figure 4.

Figure 4a presents the effect of ambient temperature on the water retention rate of the composite hydrogel. The results show that the water retention rate of the hydrogel reaches more than 93% within 72 h at room temperature of 25–30 °C. With the temperature gradually rising to 50 °C, the water retention rate slowly decreases to about 35%. From the molecular mechanism, a dense hydrogen-bonded network is formed between abundant hydroxyl/carboxyl groups and water molecules inside the hydrogel network. The stable hydrogen bonding at room temperature can strongly lock water molecules. The increase in temperature intensifies the thermal motion of water molecules, weakens the hydrogen bonding force, and accelerates water escape. Even at high temperatures, the material still maintains considerable water retention capacity, which is far superior to the pure PAA system and can adapt to the actual field environment in arid and high-temperature saline–alkali regions [24]. Analysis of variance (ANOVA) showed no significant difference in water retention of the hydrogel between the 25 °C and 30 °C treatments (*p* > 0.05). When the temperature exceeded 30 °C, the water retention decreased significantly with increasing temperature, and significant differences were observed among the 35 °C, 40 °C, 45 °C, and 50 °C groups (*p* < 0.05).

The dynamic variation of volumetric water content in soils under different treatments over time is displayed in Figure 4b. The bare soil group exhibits extremely fast water evaporation, with the water content approaching zero on day 4. The pure PAA modified group prolongs the water retention period, but only about 6% water content remains after 7 days. In contrast, the experimental group added with P(PEGMC/AA) composite hydrogel shows a higher peak soil water content and the gentlest decay, maintaining an excellent volumetric water content of more than 9% on day 7. This synergistic effect is attributed to the optimized microporous structure, increased specific surface area and uniformly distributed active functional groups. The three-dimensional porous crosslinked network constructed by PEGMC modification greatly enhances the water-locking capacity, effectively reduces ineffective evaporation of soil moisture, and significantly prolongs the soil moisture conservation period.

Figure 4c shows the cumulative leaching loss of urea in soil under different hydrogel dosages. The blank group without hydrogel has the highest urea leaching loss. As the dosage of P(PEGMC/AA) and PAA hydrogels increases from 0.05% to 0.15%, the cumulative urea loss decreases significantly, and the fertilizer control and retention effect of the P(PEGMC/AA) group is consistently better than that of the pure PAA group. At the molecular level, abundant carboxyl and hydroxyl groups on the composite hydrogel surface can form multiple hydrogen bonds and polar interactions with urea molecules, producing strong adsorption and anchoring effects on nutrient molecules, greatly inhibiting the leaching loss of fertilizer with water, realizing the synergistic long-term slow release of water and fertilizer, and improving the water and fertilizer use efficiency in saline–alkali soil [25]. Two-way ANOVA indicated that material type and additive content both exerted significant effects on the cumulative urea loss (*p* < 0.05). At 0% additive content, no significant difference was observed between P(PEGMC/AA) and PAA. Within the range of 0.05–0.15%, P(PEGMC/AA) showed significantly higher urea leakage than PAA at the same dosage. Moreover, increasing the modifier content reduced urea loss for both hydrogels.

The water absorption stability of the composite hydrogel after repeated cycles is shown in Figure 4d. After four consecutive water absorption–desiccation cycles, the P(PEGMC/AA) hydrogel still retains a water absorption ratio of nearly 200%, exhibiting excellent structural reversible recovery and cyclic durability. This is due to the dual network structure of “covalent crosslinking + reversible hydrogen bonds” in the system. Reversible hydrogen bonds can continuously break and reconstruct during drying–wetting cycles, protecting the overall covalent framework from irreversible damage and endowing the material with the potential for long-term repeated use, thus greatly reducing the practical application cost. The thin, continuous and interconnected pore wall structure in the P(PEGMC/AA) composite hydrogel can effectively disperse the internal stress generated during swelling and maintain structural integrity and durability [22]. One-way ANOVA combined with Duncan’s test (*p* < 0.05) revealed that repeated water absorption–dehydration cycles significantly influenced the water-holding capacity of the hydrogel. No significant difference was detected between the original sample (0 cycles) and the sample after one cycle. The water retention capacity decreased significantly beginning from the second cycle, and significant differences were observed among the 2nd, 3rd, and 4th cycles.

The water absorption performance of the hydrogel under different pH conditions is illustrated in Figure 4e. As the ambient pH increases from acidic (pH = 5) to neutral and weakly alkaline (pH = 5–9), the equilibrium water absorption ratio of the composite hydrogel rises steadily from 440% to 960%. Mechanistically, the carboxyl groups on the hydrogel side chains ionize in an alkaline environment, enhancing the interchain electrostatic repulsion and further stretching the polymer network, thus significantly improving the water absorption capacity. This characteristic enables it to exert better water retention and improvement effects in the weakly alkaline environment commonly found in saline–alkali soils [26]. By optimizing the crosslink density and monomer ratio, the P(PEGMC/AA) composite system maximizes the water absorption ratio in a weakly alkaline environment, providing a theoretical paradigm for designing intelligent water and fertilizer management materials for specific soil types. In practical field applications, this pH-dependent water absorption behavior also allows more precise application rates matching soil physicochemical properties. In heavily saline–alkali fields with high pH, a low dosage of P(PEGMC/AA) can achieve water retention effects comparable to high dosages of traditional materials, thereby reducing agricultural input costs and potential environmental residual risks. One-way ANOVA combined with Duncan’s multiple-range test (*p* < 0.05) demonstrated that pH significantly affected the water-holding capacity of the hydrogel. Within the pH range of 5–9, the water retention capacity increased gradually with increasing pH, and significant differences were observed between every two adjacent pH groups. Weakly alkaline conditions effectively enhanced the water absorption performance of the hydrogel.

### 2.10. Passivation and Barrier Mechanism of Composite Hydrogel on Soil Salt Ions and DFT Theoretical Verification

**Figure 5:**
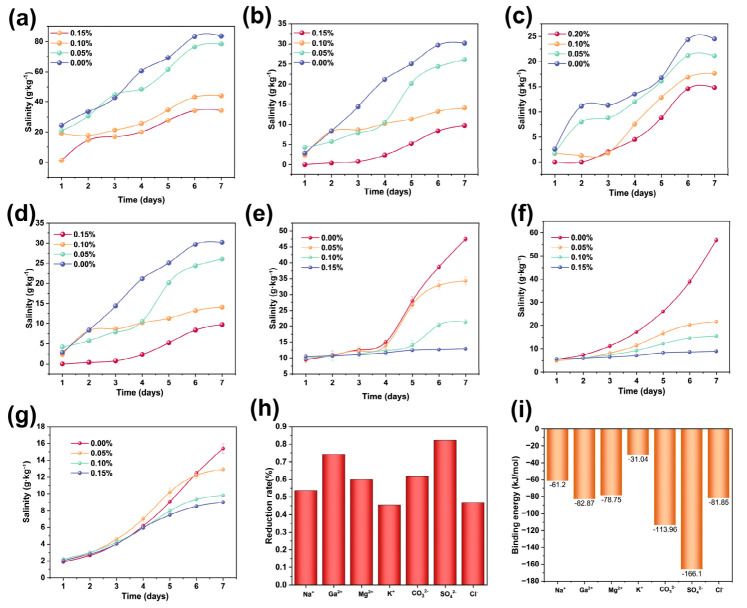
Soil salt ion immobilization effect and DFT theoretical calculation. (**a**–**g**) Dynamic changes of major soluble salt ions under different hydrogel contents; (**h**) removal efficiency of individual salt species; (**i**) DFT simulation of ion–hydrogel binding interaction energy.

Figure 5 systematically characterizes the dynamic immobilization and regulation behavior of P(PEGMC/AA) composite hydrogel on seven major soluble salt ions in saline–alkali soil (Na^+^, Ca^2+^, Mg^2+^, K^+^, CO_3_^2−^, SO_4_^2−^, Cl^−^).

Figure 5a–g show the dynamic evolution of each salt ion content with incubation period under different hydrogel application rates (0–0.15%). The results show that in the blank control group without hydrogel, all soil salt ions accumulate rapidly over time, leading to continuous aggravation of salinization. With the gradual increase of P(PEGMC/AA) hydrogel dosage, the migration and surface accumulation of all ions are significantly inhibited, and the 0.15% dosage group exhibits the optimal salt inhibition effect. The increase rate of soil ions is greatly reduced during the 7-day incubation period.

From the perspective of molecular and functional group interactions: abundant polar functional groups, including carboxyl groups (COOH), hydroxyl groups (OH) and ether bonds (C-O-C), are densely distributed on the three-dimensional network surface of P(PEGMC/AA) hydrogel. On the one hand, ionized carboxyl groups are negatively charged and can strongly capture soil cations such as Na^+^, Ca^2+^, Mg^2+^ and K^+^ through electrostatic chelation and ion coordination, restricting their upward migration with water and enrichment toward the surface layer. On the other hand, polar sites on the framework can form strong hydrogen bonds and polar adsorption with anions such as CO_3_^2−^, SO_4_^2−^ and Cl^−^, realizing simultaneous immobilization of cations and anions [27]. Meanwhile, the excellent water retention capacity of the hydrogel can uniformly regulate soil water potential, greatly weakening the capillary-driven salt accumulation effect and fundamentally delaying the process of soil secondary salinization [28,29].

The overall salt reduction rate of each ion is shown in Figure 5h. The composite hydrogel achieves the highest reduction efficiency for SO_4_^2−^ and Ca^2+^ with the most prominent inhibition effect and also exhibits excellent passivation ability for other ions, proving the broad-spectrum salt ion regulation advantage of this material.

To further clarify the microscopic nature of ion binding, density functional theory (DFT) calculations were carried out in this study. The binding energies between different ions and the hydrogel framework are shown in Figure 5i. All binding energies are significantly negative, confirming that the interactions between hydrogel and salt ions are spontaneous and stable chemisorption processes. Among them, the binding energy of SO_4_^2−^ is as low as −166.1 kJ/mol, and that of CO_3_^2−^ is −113.96 kJ/mol, showing the strongest binding effects, which are in good agreement with the macroscopic salt reduction experimental results. DFT calculations verify at the quantum chemical scale that stable coordination and strong hydrogen bonds can be formed between hydrogel functional groups and saltions, explaining the microscopic molecular mechanism of long-term salt immobilization and barrier.

### 2.11. Improvement of Saline-Alkali Soil Environment and Plant-Growth-Promoting Effect of Composite Hydrogel

**Figure 6:**
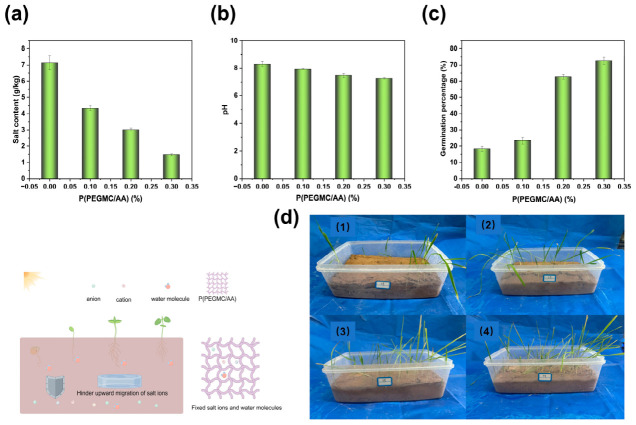
Practical desalination and plant-growth-promotion performance of P(PEGMC/AA) hydrogel. (**a**) Surface soil salinity content at different hydrogel dosages; (**b**) plant height growth under different treatments; (**c**) seed germination percentage as a function of hydrogel addition; (**d**) Representative photographs of plant germination and growth under saline conditions with P(PEGMC/AA) hydrogel dosages of 0% (1), 1% (2), 2% (3), and 3% (4), respectively. The inset illustrates the mechanism of salt upward migration inhibition, water and ion fixation by hydrogel network.

This section further verifies the practical effects of the P(PEGMC/AA) composite hydrogel on saline–alkali soil improvement and crop seedling emergence and growth, as shown in Figure 6.

As shown in Figure 6a, the topsoil salt content of the untreated control reached 7.1 g/kg. With increasing hydrogel application rate from 0 to 0.30%, soil salinity decreased continuously and significantly. At 0.30% dosage, the topsoil salt content was only 1.3 g/kg, with a desalination efficiency exceeding 80%. Mechanistically, abundant carboxyl and hydroxyl groups in the hydrogel network firmly immobilize free salt ions in soil via electrostatic chelation. Meanwhile, the 3D porous structure effectively blocks soil capillaries, greatly inhibiting the upward migration and surface accumulation of salt driven by water flow, thus fundamentally reducing salt accumulation in the plow layer and significantly alleviating soil salt stress [30]. One-way ANOVA with Duncan’s test (*p* < 0.05) showed that the P(PEGMC/AA) hydrogel significantly decreased soil salinity. Salinity was significantly higher in the control than in hydrogel treatments, and decreased significantly with increasing hydrogel dosage.

Figure 6b shows the soil pH variation under different hydrogel dosages. The original saline–alkali soil in the blank group was strongly alkaline (about pH 8.4). With the increase of hydrogel application rate, soil pH decreased gently and slightly, gradually optimizing toward the neutral range. This is because a large number of ionizable carboxyl groups on the hydrogel surface can neutralize excessive alkaline ions in soil, effectively ameliorate the alkalization characteristics of saline–alkali soil, optimize the acid-base environment, and provide suitable pH conditions for plant root growth [31,32,33]. One-way ANOVA (*p* < 0.05) indicated that soil pH slightly decreased with increasing hydrogel dosage. No significant difference was found between 0% and 0.10%, but significant differences occurred among 0.10%, 0.20% and 0.30%.

The seed germination rate is presented in Figure 6c. The seed germination rate of the high-salt blank control group was only 19%, as salt stress severely inhibited plant emergence. With the increase of hydrogel dosage, the germination rate increased significantly, reaching 75% at 0.30% application rate, nearly a four-fold improvement. On the one hand, the hydrogel effectively reduced salt toxicity and high alkali stress in soil; on the other hand, its long-term water retention capacity continuously provided a stable and suitable water environment for seed germination, greatly alleviating the dual stress damage of drought and salinity [29,34,35]. One-way ANOVA (*p* < 0.05) showed that seed germination rate increased significantly with increasing hydrogel dosage. No significant difference was observed between 0% and 0.10%, while significant differences were found among 0.10%, 0.20% and 0.30%.

The macroscopic morphology of actual plant growth in different treatment groups is intuitively displayed in Figure 6d. The plants in the blank control group were sparse and weak. In the experimental groups applied with composite hydrogel, the uniformity of seedling emergence was significantly improved, the seedlings developed roots, increased plant height and obviously enhanced biomass. Moreover, the higher the hydrogel dosage, the better the growth-promoting effect [36,37].

### 2.12. Discussion

In this study, a P(PEGMC/AA) composite hydrogel was fabricated and systematically evaluated via macroscopic tests, soil pot experiments, molecular dynamics simulations, and DFT calculations. The incorporation of PEGMC enriched the hydrogel network with carboxyl, hydroxyl, and ether polar groups, effectively improving hydrophilicity and salt resistance. Under optimal conditions, the hydrogel achieved a swelling capacity of 712 g/g in deionized water and maintained 285 g/g in a 0.9% high-salt solution. The material could form a stable hydrogen bond network to restrict water migration and immobilize saline–alkali ions (Na^+^, Ca^2+^, Mg^2+^, K^+^, CO_3_^2−^, SO_4_^2−^, Cl^−^) through electrostatic chelation, coordination, and hydrogen bonding. Pot results showed that the hydrogel ameliorated saline–alkali soil conditions, reduced water and nutrient leaching, and increased the seed germination rate from 19% to 75% under salt stress, exhibiting promising performance in water retention, slow fertilization release, salt reduction, and plant growth promotion.

Despite the promising results obtained in this study, there are still several aspects that can be further optimized in future work. First, all tests were performed under controlled laboratory conditions, and further field trials are required to verify the practical applicability and extrapolation of the research findings. Second, this study mainly focused on the short-term efficacy of the hydrogel, while its long-term structural stability, biodegradability and in-soil ecological effects need further systematic investigation. Third, the current work prioritizes material performance and mechanistic exploration, and the scale-up preparation technology and economic feasibility still need further evaluation. Fourth, the ion immobilization mechanism is elucidated based on macroscopic results and theoretical simulations, and supplementary in situ spectroscopic characterization can further consolidate the microscopic mechanism. Fifth, only single-season application effects were investigated herein, and multi-season cumulative improvement effects and soil microbial responses deserve further exploration.

Future work will address the above limitations. Long-term field trials will be conducted to verify the practical applicability and persistent remediation effect of the hydrogel. Further studies on material long-term stability, biodegradation, and soil ecological safety will be performed. Moreover, scale-up preparation optimization, economic evaluation, in situ mechanism characterization, and multi-season application tests will be carried out to supplement the current research system, thereby supporting the further field application and industrialization of the P(PEGMC/AA) hydrogel in saline–alkali soil remediation.

## 3. Conclusions

In this work, a P(PEGMC/AA) composite hydrogel was synthesized using acrylic acid and a maleic anhydride–citric acid–PEG crosslinker (PEGMC). Its preparation, swelling behavior, water and nutrient retention, salt ion immobilization, saline–alkali soil amelioration, and plant-growth-promoting effects were systematically investigated. Molecular dynamics simulations and DFT calculations were employed to reveal the underlying interaction mechanisms. The main conclusions are as follows:

Acrylic acid neutralization degree, and dosages of PEGMC crosslinker and APS initiator significantly regulate the 3D network structure and swelling performance of the hydrogel. Under optimized conditions, the composite hydrogel exhibits a swelling ratio of 712 g/g in deionized water and 285 g/g in 0.9% saline solution, showing far superior salt tolerance to pure polyacrylic acid hydrogel. At the molecular level, abundant carboxyl, hydroxyl, and ether groups form a dense hydrogen bond network with water molecules, effectively restricting water diffusion and imparting outstanding long-term water retention capacity. The hydrogel displays favorable environmental adaptability and cycling stability, effectively slowing soil water evaporation and prolonging the moisture retention period. It immobilizes urea via polar adsorption and network confinement, reducing leaching loss and realizing synchronized water and nutrient slow release. No obvious performance decay is observed under varying temperatures, pH values, and repeated drying–wetting cycles, enabling adaptation to the complex conditions of saline–alkali lands. The P(PEGMC/AA) hydrogel exhibits efficient passivation and barrier effects on seven key soil salt ions: K^+^, Ca^2+^, Na^+^, Mg^2+^, CO_3_^2−^, SO_4_^2−^, and Cl^−^. DFT calculations confirm spontaneous adsorption between gel functional groups and salt ions, achieving ion immobilization through electrostatic chelation, coordination, and hydrogen bonding, thereby effectively inhibiting topsoil salt accumulation. Pot experiments show that the composite hydrogel significantly reduces soil salinity, adjusts soil pH, alleviates saline–alkali stress, increases seed germination rate from 19% to 75%, and markedly promotes seedling growth, realizing integrated salt reduction, pH regulation, water retention, and growth promotion.

In summary, the P(PEGMC/AA) composite hydrogel has a simple synthesis route and excellent comprehensive performance, integrating water retention, salt control, nutrient slow release, and growth promotion. It exhibits promising application prospects for saline–alkali soil amelioration. Future work will focus on further formula optimization combined with long-term field experiments to facilitate its large-scale practical application.

## 4. Materials and Methods

### 4.1. Experimental Materials and Reagents

The main chemical reagents, experimental materials and soil samples used in this study are listed as follows: citric acid (CA), polyethylene glycol (PEG-2000), maleic anhydride (MA), ammonium persulfate (APS), acrylic acid (AA), anhydrous ethanol and sodium hydroxide, and they were all of analytical grade (AR), purchased from Sinopharm Chemical Reagent Co., Ltd., Beijing, China. Dialysis bags with a molecular weight cutoff of 2000 Da were obtained from Shanghai Yuanye Bio-Technology Co., Ltd., Shanghai, China. The soil samples used in the experiments were surface loess and laterite collected from Lanzhou area (sampling depth 0–20 cm). The soil samples were air-dried naturally, cleaned of impurities, ground, sieved, and then sealed for storage. Water used throughout the experiments was ultrapure water prepared in the laboratory(CA: citric acid,PEG-2000: polyethylene glycol-2000, MA: maleic anhydride, APS: ammonium persulfate, AA: acrylic acid, a poly(ethylene glycol) maleate citrate (PEGMC)).

### 4.2. Synthesis Procedures of P(PEGMC/AA) Hydrogel

#### 4.2.1. Synthesis of PEGMC

CA, PEG-2000 and MA were melted in a 250 mL three-necked round-bottom flask equipped with inlet and outlet adapters. The mixture was stirred at 160 °C for 20 min under nitrogen flow. After the components were completely melted, the temperature was lowered to 145 °C and maintained for 2 h. Subsequently, the pressure was reduced to 50 mTorr and held for 2 h. The as-prepared prepolymer was dissolved in deionized water, dialyzed against a 2000 Da molecular weight cutoff membrane for 2 days, and then freeze-dried to obtain purified PEGMC. In the initial composition of the prepolymer, the acid ratio (MA/CA) was adjusted to 4/6, respectively. The overall molar ratio of total acids to diol was kept at 1:1 [13].

#### 4.2.2. Synthesis of P(PEGMC/AA) Hydrogel

**Figure 7:**
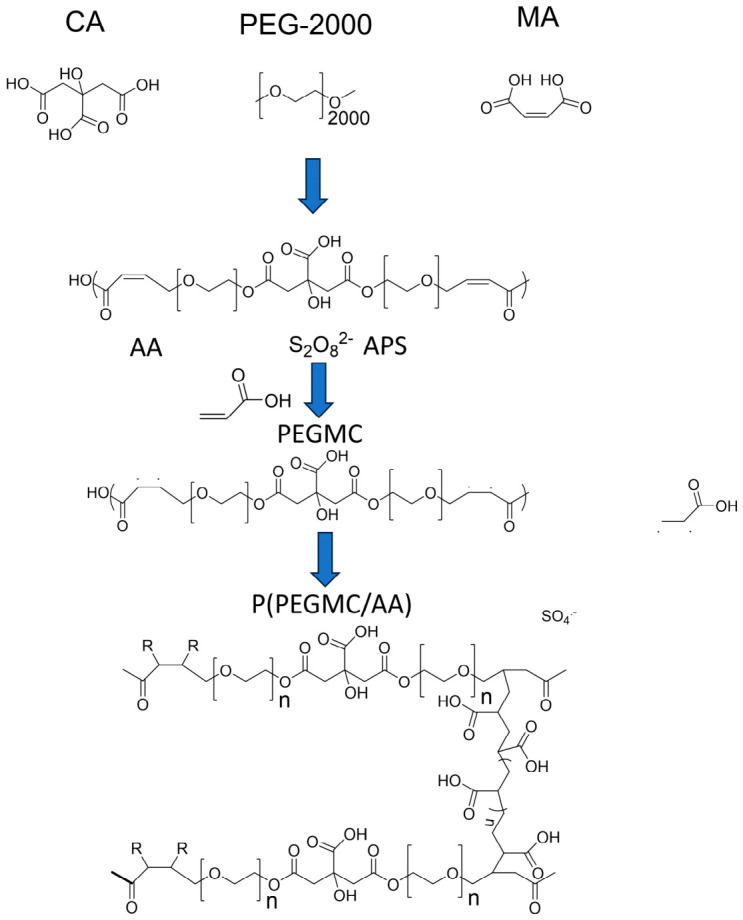
Schematic illustration for the synthesis mechanism of PEGMC crosslinker and P(PEGMC/AA) hydrogel.

The synthesized PEGMC was added into a four-necked flask, followed by 15 mL of deionized water. The mixture was stirred at 300 r/min and 45 °C and purged with nitrogen for 15 min. After adding the thermal initiator and reacting for 30 min, AA was introduced, and the system was kept at 65 °C for 2 h under continuous nitrogen protection. The obtained gel was soaked in anhydrous ethanol for 6 h, then transferred to an oven at 80 °C for three days, and subsequently ground into powder. The 40-mesh powder was used for experiments, while the 200-mesh powder was used for characterization. The reaction mechanism is depicted in Figure 7.

### 4.3. Material Characterization

Fourier transform infrared spectroscopy (FT-IR spectrometer (Digilab LLC, Marlborough, MA, USA)) was used to characterize the chemical structures of the monomers MA, CA, and PEG, the homopolymer PAA, the copolymer P(PEGMC/AA), and the crosslinker PEGMC. Tests were performed in attenuated total reflection (ATR) mode, with a scanning wavenumber range of 4000–500 cm^−1^ and a resolution of 4 cm^−1^. Each sample was scanned 32 times cumulatively to improve the signal-to-noise ratio. Before testing, solid samples were fully dried and pressed firmly onto the ATR crystal; liquid samples were directly dropped onto the testing area, and spectra were collected after the baseline stabilized. (All gels were tested as 200-mesh powders.)

Thermal stability tests were carried out on a thermogravimetric analyzer (TGA, Shimadzu, Kyoto, Japan). Approximately 5–10 mg of dried PAA and P(PEGMC/AA) xerogel samples were weighed and placed in clean alumina crucibles. Tests were performed under a high-purity nitrogen atmosphere at a flow rate of 50 mL/min. The temperature was increased from room temperature (25 °C) to 800 °C at a constant heating rate of 10 °C/min. The variation in sample mass with temperature was recorded in real time to analyze the thermal decomposition behavior and differences in thermal stability (All gels were tested as 200-mesh powders).

The surface micromorphology of the samples was observed using a scanning electron microscope (SEM, Carl Zeiss AG, Oberkochen, Germany). Before testing, PAA and P(PEGMC/AA) gel samples were brittle-fractured in liquid nitrogen to obtain fresh cross-sections, followed by complete dehydration via vacuum freeze-drying. The dried samples were mounted on sample stubs and sputter-coated with gold using an ion sputtering coater to improve conductivity. The surface morphology and cross-sectional structure of the samples were observed and recorded at an accelerating voltage of 5–10 kV, and the pore structure and surface characteristics were compared (all gels were tested as 200-mesh powders).

P(PEGMC/AA) gel samples were cast onto Petri dishes and dried in an oven at 60 °C. A small amount of the dried sample was uniformly dispersed in anhydrous ethanol and sonicated for 10 min to achieve full dispersion. A small amount of the suspension was pipetted onto a freshly cleaved mica substrate and air-dried naturally before atomic force microscopy (AFM) testing. The tests were performed in air at room temperature using tapping mode. The scanning range was set to an appropriate scale according to the sample morphology, and the scanning rate was set to 1.0 Hz. The obtained topography images were processed using the built-in analysis software to obtain the surface roughness, height distribution, and three-dimensional morphology of the samples, thereby characterizing the micro-surface morphology and structural uniformity of the gel.

### 4.4. Performance Testing of P(PEGMC/AA) Material

#### 4.4.1. Water Absorption Test

A total of 0.1 g of P(PEGMC/AA) material was weighed and immersed in 500 mL of deionized water, tap water, and 0.9 wt% NaCl aqueous solution, respectively, and soaked for 4 h to reach swelling equilibrium. The sample was filtered and weighed, and the water absorption capacity was calculated (three parallel experiments were conducted).

Water absorption equation:

(1) $$Q_{eq}\;=\;\frac{\left(M_{1}-M_{0}\right)}{M_{0}}$$

$M_{1}$ indicates the weight of P(PEGMC/AA) material after water absorption, $M_{0}$ and P(PEGMC/AA) material [38].

#### 4.4.2. Repeated Swelling Behavior and Water Absorption at Different pH Values

A total of 0.1 g of P(PEGMC/AA) material was soaked in 500 mL of deionized water for 4 h to reach swelling equilibrium, filtered, weighed, and then dried in an oven at 60 °C. After drying, the above procedure was repeated for 5 successive swelling–drying cycles (three parallel experiments were conducted).

The water absorption of the material at different pH values was measured separately and calculated as follows:

(2) $$W_{h}\left(\%\right)=\frac{\left(M_{1}-M_{0}\right)}{M_{0}}*100\%$$

$M_{1}$ indicates the weight of P(PEGMC/AA) material after water absorption, and $M_{0}$ indicates the weight of P(PEGMC/AA) material [38].

#### 4.4.3. Water Retention at Different Temperatures

P(PEGMC/AA) material was immersed in 500 mL of deionized water and soaked for 4 h to reach swelling equilibrium. At 25 °C, 30 °C, 35 °C, 40 °C, 45 °C, and 50 °C, 100 g of the swollen sample was weighed once per hour for 12 consecutive measurements, and the water retention rate $W_{r}\;(\%)$ was calculated (three parallel experiments were conducted).

Water retention equation:

$$W_{r}\;(\%)=(M_{n}/M_{0})*100\%$$

$M_{n}$ represents the weight of each weighing and $M_{0}$ the original weight [24].

#### 4.4.4. Long-Term Mechanism of Hydrogels in Soil Moisture Conservation and Inhibiting Water Evaporation

Separately, 250 mL of water, 250 g of PAA hydrogel, and 250 g of P(PEGMC/AA) hydrogel were mixed with soil, stirred uniformly, and placed in an outdoor environment. The soil water content was measured at 20:00 every night for 7 consecutive days.

A portable digital soil temperature and moisture meter was used for in situ measurement of soil volumetric water content at fixed points. During the experiment, the probe was inserted vertically into each soil sample at the same depth at a fixed time each day, and the volumetric water content was recorded after the reading stabilized. Three parallel measurement points were set for each group, and the average value was taken as the daily water content. Dynamic curves of soil water retention capacity over time for different treatment groups were plotted (three parallel experiments were conducted).

#### 4.4.5. Fertilizer Maintenance

To investigate the effect of P(PEGMC/AA) material on soil fertilizer leaching, the material was mixed thoroughly with 250 g of loess and 0.3 g of urea at addition ratios of 0.0%, 0.05%, 0.10%, and 0.15%. The mixture was packed into a PVC plastic column with a diameter of 8 cm. A piece of wet filter paper was placed at the bottom of the column, followed by another layer of filter paper and 25 g of quartz sand on top.

A total of 200 mL of deionized water was added to the soil column for leaching, and the leachate was collected every 3 days. Deionized water was replenished after each 3-day interval, and this process was repeated 5 times. The urea concentration in the leachate was determined using a using a UV-Vis spectrophotometer (Model UV-2800, UNICO Instruments Co., Ltd., Shanghai, China).

First, *p*-dimethylaminobenzaldehyde color reagent and urea were mixed at a volume ratio of 1:1, and scanning was performed over the wavelength range of 200–800 nm to determine the maximum absorption wavelength. Then, urea standard solutions of different concentrations were prepared, mixed with an equal volume of color reagent in colorimetric tubes, and diluted to a constant volume of 25 mL. A calibration curve was established by plotting absorbance against urea concentration. Finally, the leachate was mixed with the color reagent, and the urea concentration was measured (three parallel experiments were conducted).

#### 4.4.6. Salt Isolation

P(PEGMC/AA) material was uniformly mixed with 500 g of saline–alkali soil at addition ratios of 0.0%, 0.05%, 0.10%, and 0.15%. Filter paper was placed at the bottom of a PVA plastic cylinder with a radius of 4 cm, followed by 250 g of quartz sand. The above mixed saline–alkali soil was then added, and the upper layer was covered with 500 g of laterite. A salt solution with a concentration of 3 g/L was introduced into the column.

A 250 W lamp was used to simulate sunlight irradiation for 12 h, after which surface soil samples were collected. The above procedure was repeated daily until the salt content in the surface layer stabilized.

After drying, 3 g of surface soil was weighed, ground, sieved, placed in a microwave digestion vessel, and mixed with 65% nitric acid and 40% hydrofluoric acid. Digestion was performed in a microwave digester (Model RWX-50, Shandong Youyunpu Photoelectric Technology Co., Ltd., Weifang, China). After digestion, the solution was transferred to a beaker, heated in a water bath and boiled for 1 h, then diluted to 25 mL with deionized water and mixed uniformly. After centrifugation, 1 mL of the supernatant was diluted, filtered through a 0.22 μm needle filter, transferred to a 10 mL plastic tube, and the concentrations of K^+^, Ca^2+^, Na^+^, and Mg^2+^ were determined using an inductively coupled plasma mass spectrometer (ICP-MS, NexION 350D, PerkinElmer Inc., Waltham, MA, USA) (Three parallel experiments were conducted).

#### 4.4.7. Planting

P(PEGMC/AA) material was mixed with saline–alkali soil at mass fractions of 0.0%, 0.1%, 0.15%, and 2%. The soil was placed in plastic culture boxes (45 cm in length × 30 cm in width). Sixty oat seeds were sown uniformly in each box, 100 mL of deionized water was supplied daily, and seed germination was recorded. After 3 weeks, the electrical conductivity and pH of the surface soil at a depth of 2 cm were measured (three parallel experiments were conducted).

Germination rate:

$$G(\%)=N_{0}/N*100\%$$

where $G\left(\%\right)$ is the germination rate, $N_{0}$ is the number of germinated seeds, and *N* is the total num ber of tested seeds [38].

## Data Availability

The original contributions presented in this study are included in the article. Further inquiries can be directed to the corresponding author.
